# Religious service attendance and mortality: A population-based prospective cohort study in southern Sweden

**DOI:** 10.1016/j.ssmph.2023.101492

**Published:** 2023-08-17

**Authors:** Martin Lindström, Mirnabi Pirouzifard

**Affiliations:** Social Medicine and Health Policy, Department of Clinical Sciences and Centre for Primary Health Care Research, Lund University, S-205 02, Malmö, Sweden

**Keywords:** Religious service attendance, Religion, Generalized trust in other people, Social capital, Health-related behaviors, Cardiovascular mortality, Cancer mortality, Sweden

## Abstract

**Aims:**

The aim is to investigate associations between attendance in religious service during the past year and all-cause, cardiovascular (CVD), cancer and other cause mortality.

**Study design:**

Prospective cohort study.

**Methods:**

A public health survey with three reminders was sent to a stratified random sample of the adult 18–80 population in southernmost Sweden in 2008. The response rate was 54.1%, and 24,855 participants were included in this study. The cross-sectional baseline survey was connected to mortality data with 8.3-year follow-up. Analyses were conducted in Cox regression models.

**Results:**

13.9% had attended religious service at least once during the past year, and 86.1% had not attended. The group with religious attendance contained significantly higher proportions of women, high and medium position non-manual employees, participants born abroad, never alcohol consumers, respondents with high trust in others and respondents with high social participation. It also contained significantly lower proportions with low leisure-time physical activity (LTPA) and daily smokers. Religious service attendance during the past year was significantly associated with lower hazard rate ratios (HRRs) of all-cause mortality compared to non-attendance until social participation items were introduced in the final model. HRRs of CVD mortality were significantly lower for religious attendance in the multiple models until BMI and health-related behaviors were introduced. No significant results were observed for cancer and other cause mortality.

**Conclusions:**

The results suggest that religious service attendance in a highly secularized country such as Sweden is significantly associated with lower all-cause mortality, which may be explained by a social network pathway in this highly secularized population.

## Highlights

• Religious service attendance and mortality have mostly been investigated in the US.
• Studies from more secularized countries in Europe such as Sweden are very scarce.
• A stratified random sample of the population aged 18–80 in southern Sweden was investigated.
• Religious service attendance during the past year was associated with lower mortality.
• Lower CVD mortality was a major contributor, partly mediated by health-related behaviors.

## 1 Introduction

Religion is broadly regarded as a social determinant of health (Idler, 2014). Religion may encourage and support helpful and health promoting practices and benevolent health-related behaviors (Chen & WanderWeele, 2018). Two additional major causal pathways between religion and health have also been suggested and investigated. One pathway includes collective organized religiosity as well as more individual spirituality as means of psychological coping with psychosocial adversity and stress (George et al., 2002). The second suggested pathway includes wellbeing through social connections and social participation in religious service attendance, and the social support that stems from such social connections and social participation. (Kawachi, 2019).

Religion, religious service attendance and prayer are associated with psychological health. Religious service attendance is for example associated with lower risk of depression (Shanshan et al., 2016) and lower suicide rates (VanderWeele et al., 2016) among US women. Concerns regarding reverse causality in cross-sectional studies have been raised because some participants are prone to stop religious attendance after the onset of major depression (Maselko et al., 2012). Still, these concerns may primarily be valid with regard to cross-sectional studies. The possibility remains that religious experiences and sense of a higher meaning connected with religious service attendance may promote a sense of peace, hope, and an existentially deeper sense of meaning in life (Koenig, 2014).

The second suggested pathway concerns religious service attendance as a form of social participation and source of social support. Social participation in social activities and organizations such as for instance social participation in organized religion is a key characteristic of social capital (Lindström & Rosvall, 2019). Social participation in a general sense is a central feature of social capital both within the “cohesion” approach (Coleman, 1990; Putnam, 1993) and within the “network” approach to social capital (Bourdieu, 1986). Pathways between social capital and health defined as social participation/network, generalized trust in other people and reciprocity within the “cohesion” approach to social capital (Putnam, 1993) include psychological and psychosocial stress mechanisms, norms regarding health-related behaviors, access to healthcare and other amenities, and reduced exposure to violent crime (Kawachi et al., 1999).

The health outcomes in this study are all-cause and cause-specific mortality obtained from Swedish official register data. Previous international studies have investigated associations between religious service attendance/religious involvement and mortality finding significant associations between religious service attendance and/or religious involvement and lower all-cause mortality (Chida et al., 2009; Gillum et al., 2008; Hummer et al., 1999; Li et al., 2016; McCullough et al., 2000; Musick et al., 2004). However, only few studies have included cause-specific mortality. One US study found significant associations between religious service attendance and both cardiovascular disease (CVD) and cancer mortality (Li et al., 2016). Most studies have been conducted in the US. Fewer studies have been conducted in northern and Western Europe (Ahrenfeldt et al., 2023; Ralston et al., 2017; Vanderweele, 2017). A recent Danish study showed lower mortality among participants attending religious service at least once a month, but only for women (Ahrenfeldt et al., 2023). Sweden is considerably more secularized than the US and somewhat more secularized than Denmark. It would thus be of high interest to investigate associations between religious service attendance and mortality in a secularized and individualized high-income country and setting such as Sweden.

Classic sociologist Émile Durkheim was the first to display the importance of social context for the association between religion and health in “Suicide” (1897). Suicide rates were much higher among minority protestant Christians in Catholic southern Germany than among majority protestant Christians in northern Germany (Durkheim, 1951/1897). The significance of such social contexts for the health of religious minorities may be just as important when the majority population consists of secularized irreligious people. Atheists and agnostics also have convictions and beliefs that may lead to sentiments ranging from indifference to animosity towards religious minorities and believers in God in the context of an overwhelmingly secularized society. The Soviet Union (1917–1991), its satellite countries as well as other Communist states are extreme cases of even state-directed criminalization and persecution against religion.

From 1972 to 2000, membership in the Lutheran Swedish State Church declined comparatively slowly from 7.8 million (95.2% of the total population) to 7.4 million (82,9%), but from 2000 when the Lutheran Church was separated from the state membership decline accelerated to 5.7 million (55.2%) in 2020. In recent decades the Lutheran Church has been losing 1% of its members every year and many remaining never attend religious services. In the late 2010s, approximately 500,000 persons in Sweden self-identified as Muslims and 20,000 as Jews. Approximately 800,000–900,000 persons belong to recognized religious congregations, churches and organizations outside the Lutheran Swedish church, including immigrant Christians, Muslims, Jews, Swedish free churches, the Catholic Church and other denominations. This means that of a total population of 10.4 million in the late 2010s, a large and growing proportion of the total population has no membership in neither the Lutheran Swedish church nor any other congregation of any other denomination. In this context, Sweden is not only highly secularized in international comparison but also highly individualized according to the World Values Survey (WVS) (Inglehart, 2018), because the Swedish population is subjected to state individualism to a considerably higher extent than probably any other population in the world. The dependence in Sweden of the individual citizen on the state for access to welfare amenities and support has replaced traditional intermediate social structures such as church, family and voluntary civil associations and organizations. Furthermore, previous studies have typically not investigated other crucial characteristics of social capital such as generalized trust in other people in studies concerning religious service attendance and mortality, despite the fact that trust may be associated with both religion and culture (Inglehart, 1997). We also include various aspects of social participation in the final multiple model, adjusting for the social network pathway between religious service attendance and mortality.

The aim is to investigate associations between attendance in religious service during the past year and all-cause, CVD, cancer and other cause mortality, adjusting for relevant covariates including generalized trust in others and various aspects of social participation.

## 2 Material and methods

### 2.1 Study population

In the autumn of 2008, a cross-sectional public health survey based on a stratified sample of the population aged 18–80 was conducted. A letter of invitation with a questionnaire was sent by post, followed by three postal reminders to initial non-respondents. It was also possible to answer the questionnaire online. The response rate was 54.1%. The survey data include in total 28,198 respondents. *Region Skåne*, the regional authority responsible for the healthcare system in the southernmost part of Sweden, funded and conducted the public health survey. The questionnaire entails more than 130 items regarding self-reported general and psychological health (GHQ12), social support, social participation, generalized trust in other people, institutional trust, psychosocial and physical work conditions, health-related behaviors, security and sociodemographic items. The random sample was stratified into 60 geographical areas (municipalities and city parts) by age, sex and education to obtain statistical power in each geographical unit. *Statistics Sweden* created the stratified sample, and the population weight compensating for the stratification in this study. The population weight takes into account lower response rate among men, younger people, people born abroad and people with less education. The cross-sectional baseline 2008 survey data was connected to prospective mortality data from *Socialstyrelsen*.

The present study was approved by the Ethical Committee (*Etikprövningsnämnden*) in Lund (No. 2010/343).

### 2.2 Dependent variables

Mortality was followed from 27 August-14 November 2008 (according to registration date of individual answers) until December 31, 2016 (8.3 years onwards), or until death. A total of 24,855 participants were included in this study, excluding respondents with internally missing values on any or several of the variables analyzed in this study, and 136 respondents lost to follow-up. Causes of death were registered according to ICD10. The Swedish ten-digit person number system makes linkage of baseline data from the 2008 survey with the Swedish national causes of death register at the Swedish *Socialstyrelsen* by a third party (private company) possible. The ten-digit person numbers were erased before delivery from the *Socialstyrelsen* to the researchers.

All-cause (total), CVD (I00–I98), cancer (C00–C97), and all other causes (than I00–I98 and C00–C97) mortality were analyzed. All-cause mortality is the sum of the other three cause-specific categories.

### 2.3 Independent variables

*Religious service attendance during the past year* was assessed with the question “Have you attended church/religious service during the past year”. This item is a part of a social participation item that concerns whether or not the respondent has taken part in formal and informal groups and social activities during the past year. The 13 activities include study circle/course at work, other study circle/course, union meeting, other meeting, theatre/cinema, arts exhibition, church/religious service attendance, sports event, letter to editor of newspaper/journal, demonstration, night club/entertainment, big gathering of relatives and private party. A fourteenth alternative was none of the above.

**Fig. 1:**
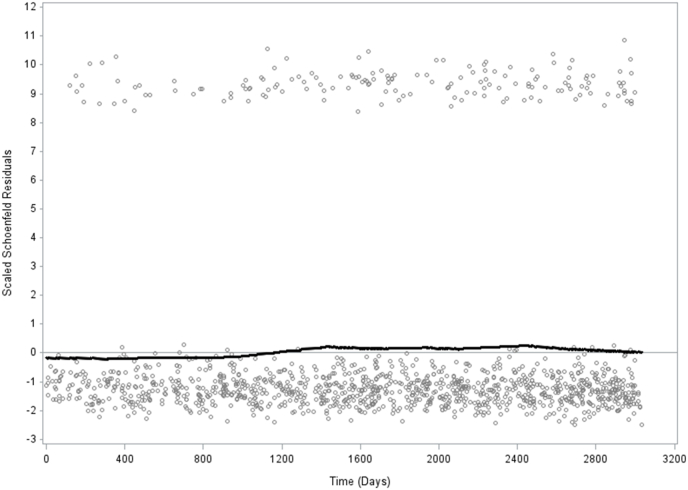
Schoenfeld residuals for men and women according to religious service attendance and all-cause mortality over the 8.3-year period 2008–2016. The proportionality test based on interaction between religious service attendance and time with regard to all-cause mortality over the 8.3-year follow-up time showed p = 0.194, which indicates proportionality.

Men and women were collapsed in all analyses in Table 1, Table 2 and Fig. 1. Analyses in Table 2 were adjusted for sex (and all other covariates).

**Table 1:** Descriptive characteristics (mean) of age and BMI, and descriptive statistics (%) of sex, SES, country of birth, chronic disease, low leisure-time physical activity (LTPA), daily smoking, alcohol consumption, generalized trust on other people and twelve social participation items by religious service attendance. The 2008–2016 Public Health Survey of Scania, Sweden. Total population **n=24855** (11300 men and 13555 women). **Weighted prevalence.**

	Religious service attendance at least once during the past year		
	No (0)	Yes (1)	p-value
	n = 21093 86.08%	n = 3762 13.92%	
**Death (n)**	1098	157	0.008
**Age**, yrs: mean ± SD a	45.83 ± 16.81 (45.53–46.12)	46.20 ± 16.33 (45.50–46.89)	0.218
**BMI:** mean ± SD a	25.56 ± 4.55 (25.48–25.64)	25.48 ± 4.25 (25.30–25.65)	0.322
**Sex**^b^			
Male	51.21 (50.38–52.05)	43.05 (41.07–45.03)	<0.001
Female	48.79 (47.95–49.62)	56.95 (54.97–58.93)	
**Socioeconomic status (SES)**^b^			<0.001
High non-manual	8.47 (8.01–8.93)	12.68 (11.37–13.99)	
Medium non-manual	13.55 (12.99–14.10)	17.16 (15.75–18.57)	
Low non-manual	8.12 (7.67–8.58)	7.35 (6.29–8.41)	
Skilled manual	11.07 (10.54–11.60)	7.22 (6.20–8.24)	
Unskilled manual	12.94 (12.38–13.50)	10.44 (9.06–11.82)	
Self-employed/farmer	6.07 (5.67–6.46)	5.91 (4.92–6.90)	
Early retired	3.87 (3.53–4.22)	3.17 (2.47–3.87)	
Unemployed	3.89 (3.52–4.26)	3.90 (2.99–4.81)	
Student	8.21 (7.68–8.74)	8.96 (7.63–10.30)	
Old age pensioner	16.99 (16.41–17.57)	18.30 (16.88–19.73)	
Unclassified	5.67 (5.22–6.11)	3.82 (2.93–4.71)	
Long-term sickleave	1.15 (0.97–1.34)	1.08 (0.65–1.51)	
**Born outside Sweden**^b^	16.98 (16.24–17.73)	21.38 (19.52–23.23)	<0.001
**Chronic disease**^b^	28.38 (27.63–29.13)	28.81 (27.08–30.54)	0.295
**Low leisure-time physical activity (LTPA)**^b^	14.22 (13.59–14.85)	11.15 (9.78–12.53)	<0.001
**Daily smoking**^b^	15.22 (14.61–15.83)	8.36 (7.11–9.60)	<0.001
**Alcohol drinking past year**^b^			<0.001
Never	10.50 (9.94–11.07)	16.34 (14.79–17.88)	
Once a month or more seldom	22.89 (22.18–23.60)	22.24 (20.52–23.97)	
2–4 times a month	36.64 (35.80–37.49)	32.30 (30.39–34.21)	
2–3 times a week	22.63 (21.94–23.33)	22.45 (20.76–24.14)	
At least 4 times a week	7.33 (6.93–7.74)	6.67 (5.81–7.53)	
**Low generalized trust in other people**^b^	37.58 (36.71–38.45)	30.01 (28.21–31.81)	<0.001
**Study circle/course at work**	23.04 (22.35–23.72)	31.25 (29.34–33.16)	<0.001
**Other study circle/course**	14.30 (13.72–14.88)	26.29 (24.46–28.13)	<0.001
**Union meeting**	8.02 (7.59–8.46)	11.47 (10.24–12.69)	<0.001
**Other meeting**	22.87 (22.14–23.59)	44.34 (42.29–46.39)	<0.001
**Theatre/cinema**	61.60 (60.79–62.40)	74.50 (72.69–76.31)	<0.001
**Arts exhibition**	28.86 (28.13–29.60)	47.58 (45.52–49.63)	<0.001
**Sports event**	42.36 (41.54–43.18)	46.18 (44.18–48.18)	<0.001
**Letter to editor of newspaper/journal**	4.35 (3.98–4.71)	7.16 (6.13–8.17)	<0.001
**Demonstration**	2.79 (2.47–3.10)	3.82 (3.04–4.61)	<0.001
**Night club/entertainment**	47.89 (47.03–48.75)	48.62 (46.68–50.56)	0.429
**Big gathering of relatives**	47.72 (46.90–48.54)	67.73 (65.86–69.60)	<0.001
**Private party**	82.17 (81.51–82.83)	89.28 (87.99–90.58)	<0.001

The values in parentheses are 95% confidence intervals for mean or percent based on bootstrap method with 1000 number of replicates.

a p-value: *t*-test, 2-tailed.

b p-value: Pearson Chi Square test, 2-sided.

**Table 2:** Hazard rate ratios (HRRs) with 95% confidence intervals (95% CIs) of all-cause, CVD, cancer and other cause mortality according to religious service attendance during the past year. The 2008–2016 Scania public health survey with 8.3 years follow-up. All n = 24855 (11300 men and 13555 women). **Weighted prevalence.**

Cause of death	Model 0		Model 1		Model 2		Model 3		Model 4		Model 5		Number of deaths
	HR	(95%CI)	HR	(95%CI)	HR	(95%CI)	HR	(95%CI)	HR	(95% CI)	HR	(95% CI)	
**All causes**													
Non-religious	1.00		1.00		1.00		1.00		1.00		1.00		
Religious	0.82	(0.67–1.01)	0.75**	(0.62–0.92)	0.76**	(0.62–0.93)	0.81*	(0.66–0.99)	0.81*	(0.67–0.99)	0.89	(0.73–1.09)	1255
**Cardiovascular disease**													
Non-religious	1.00		1.0		1.00		1.00		1.00		1.00		368
Religious	0.69*	(0.47–0.99)	0.65*	(0.44–0.94)	0.65*	(0.45–0.95)	0.69	(0.47–1.01)	0.69	(0.47–1.01)	0.75	(0.51–1.13)	
**Cancer**													
Non-religious	1.00		1.00		1.00		1.00		1.00		1.00		499
Religious	0.85	(0.61–1.19)	0.77	(0.55–1.07)	0.77	(0.55–1.08)	0.83	(0.60–1.17)	0.84	(0.60–1.18)	0.89	(0.63–1.24)	
**Others**													
Non-religious	1.00		1.00		1.00		1.00		1.00		1.00		
Religious	0.92	(0.65–1.31)	0.85	(0.59–1.21)	0.85	(0.60–1.21)	0.89	(0.64–1.25)	0.90	(0.64–1.26)	1.03	(0.72–1.48)	388

Model 0 unadjusted.

Model 1 adjusted for sex and age.

Model 2 additionally adjusted for socioeconomic status, country of birth and chronic disease.

Model 3 additionally adjusted for BMI, leisure-time physical activity, smoking and alcohol consumption.

Model 4 additionally adjusted for generalized trust in other people.

Model 5 additionally adjusted for study circle/course at work, other study circle/course, union meeting, other meeting, theatre/cinema, arts exhibition, sports event, letter to the editor of newspaper/journal, demonstration, night club/entertainment, big gathering of relatives and private party, all at least once during the past year.

Significance levels: *p < 0.05, **p < 0.01, ***p < 0.001.

Weighted Hazard Ratios. Bootstrap method (1000 replicates) for variation estimation.

*Age* was treated as a continuous variable in the analyses.

*Country of birth* was dichotomized into born in Sweden or other country.

*Socioeconomic status (SES)* (by occupation and labor market) was defined as non-manual employees in high, medium and low positions, skilled and unskilled manual workers, and self-employed/farmers. Groups outside the workforce include unemployed, students, early retired (before 65), long-term sick leave, pensioners aged 65-, and unclassified.

*Chronic disease* was measured with the question “Do you have any long-term disease, ailment or injury, any disability or other weakness?”, with the alternatives “Yes” and “No”.

*Body mass index (BMI)* was analyzed as a continuous variable.

*Leisure-time physical activity* (LTPA) was assessed with the four alternatives regular exercise (at least three times per week at least 30 min/occasion, leading to sweating), moderate regular exercise (exercising once or twice per week at least 30 min/occasion, leading to sweating), moderate exercise (walking, cycling or equivalent activity status in leisure-time less than 2 h walking, cycling or equivalent activity/week) and low LTPA (less than 2 h walking, cycling or equivalent activity/week). The LTPA item was dichotomized, with the fourth alternative defined as low LTPA.

*Daily smoking* was assessed with the item “Do you smoke?” with the alternatives daily, non-daily and non-smoker, collapsing the two latter alternatives.

*Alcohol consumption* was measured with the question “How often have you consumed alcohol during the past twelve months?” with alternatives “4 times per week or more”, “2–3 times per week”, “2–4 times per month”, “Once per month or more seldom”, and “Never”.

*Generalized trust in other people* was obtained with the question “Most people can be trusted”, with alternatives “Do not agree at all”, “Do not agree”, “Agree” and “Agree completely”, with the two first alternatives collapsed as “low trust” and the two latter “high”.

The other 12 *social participation* items include study circle/course at work, other study circle/course, union meeting, other meeting, theatre/cinema, arts exhibition, sports event, letter to editor of newspaper/journal, demonstration, night club/entertainment, big gathering of relatives and private party at least once during the past year.

### 2.4 Statistics

Prevalence (%) of all variables were analyzed based on the two categories religious service attendance during the past year or not. Differences between the two categories were analyzed using *t*-test for continuous variables and chi-square test for categorical variables (p-values) (Table 1). Hazard rate ratios (HRR:s) with 95% confidence intervals (95% CI:s) of all-cause, CVD, cancer and other causes mortality by religious service attendance were calculated. Five Six models were calculated: model 0 unadjusted, model 1 adjusted for sex and age, model 2 adjusted for sex, age, country of birth, SES, and chronic disease, model 3 additionally adjusted for BMI, LTPA, daily smoking and alcohol consumption, model 4 additionally adjusted for generalized trust in other people, and model 5 additionally adjusted for the social participation items (Table 2). Follow-up was assessed as days from baseline 2008 to death or last follow-up date (December 31, 2016). Investigation of sampling variability without distributional assumptions regarding the study population are possible with bootstrap analysis (SAS/STAT Software Survey Analysis, 2021). Bootstrap methods with 1000 numbers of replicates to make accurate variance estimation on weighted data were used to obtain confidence intervals and p-values. Test of proportionality for religious attendance and mortality was conducted. The assumption of proportional hazards was assessed with an interaction term with time and religious attendance. Schoenfeld residuals were calculated for religious attendance (yes/no) and mortality (Fig. 1). The SAS software version 9.4 was utilized for the calculations.

## 3 Results

Table 1 shows that the group that had attended religious service during the past year (13.92%) were women, non-manual employees in higher and medium positions, foreign-born, not daily-smokers, never-consumers of alcohol and had high generalized trust in other people to a significantly higher extent than respondents in the group that had not attended religious service (86.08%) during the past year. The group of religious service attenders had also participated to a significantly higher extent in most aspects of social participation during the past year compared to non-attenders. The group that had attended religious service during the past year also had a significantly lower proportion of respondents that reported low LTPA. In contrast, no statistically significant differences were observed regarding age, BMI and chronic disease between the two groups.

Table 2 shows that the group that attended religious service during the past year had significantly lower hazard rate ratios (HRRs) of all-cause mortality compared to the group that did not attend religious service during the past year until social participation items were added in multiple model 5. In the age- and sex-adjusted model 1, a HRR 0.75 (0.62–0.92) was observed, and in model 4 a statistically significant HRR 0.81 (0.67–0.99) of all-cause mortality was observed for religious service attendants compared to the group that had not attended. In the final model 5, a HRR 0.89 (0.73–1.09) was observed after adjustment for all twelve social participation items. The religious service attendance group had lower HRRs of CVD mortality in models 0–2 compared to the non-attendance group. A significant HRR 0.65 (0.45–0.95) of CVD mortality was still observed in model 2, but changed to not statistically significant HRR 0.69 (0.47–1.01) in model 3 and HRR 0.75 (0.51–1.13) in model 5. No statistically significant differences in cancer and other cause mortality between the group that had attended religious service during the past year compared to the group that had not attended were observed in models 0–5.

Fig. 1 shows that Schoenfeld residuals for religious attendance versus non-attendance and all-cause mortality were consistent and stable over 8.3-years follow-up. The interaction term between religious attendance and time with regard to all-cause mortality across the 8.3-year period showed a statistically not significant p = 0.194, which indicates proportionality.

## 4 Discussion

Religious service attendance during the past year was significantly associated with lower HRRs of all-cause mortality compared to non-attendance until the social participation items were introduced in the final model. Lower CVD mortality was a major contributor to this consistent pattern, because HRRs of CVD mortality were significantly lower for religious attendance in the multiple models until BMI, LTPA, daily smoking and alcohol consumption were introduced in model 3. It seems that significantly lower prevalence of low LTPA, daily smoking and alcohol consumption may contribute to some extent to lower CVD mortality in the religious attendance group. No significant associations between religious attendance during the past year and cancer and other cause mortality were observed. The main results that religious service attendance is significantly associated with lower mortality before social participation items were introduced confirm results from previous studies mainly from the US, a less secularized country with considerably higher religious service attendance than Sweden.

To our knowledge, no previous study has adjusted for other forms of social participation to account for the plausible social network pathway between religious service attendance and mortality. The adjustments for various aspects of social participation than religious service attendance may to an important extent account for the social network pathway between religious service attendance and mortality. However, it should be noted with caution that the 2008 public health questionnaire that forms the baseline of this study does not contain any other item on religious activities or information regarding denominational belonging than the religious service attendance at least once a year item analyzed as exposure in this study. The recently published Danish study is based on religious service attendance at least once a month (Ahrenfeldt et al., 2023), while the international literature mostly uses the cut-off at least once a week to denote moderate religiosity. In our study, a total 13.9% had attended religious service at least once a year, while in the Danish study a total 5.0% of men and 6.6% of women had taken part in a religious organization at least once a month. Since Sweden is a somewhat more secularized country than Denmark, it is highly probable that substantially less than 5.0%–6.6% would have attended religious service at least once a month in our study population. Our study results are thus probably diluted by participants who very rarely (although at least once a year) attend religious service. The conclusion that the social network pathway between attendance and mortality is most important for health may not be applicable to the smaller fraction of religious service attenders who attend religious service at least once a week. Spirituality and deeper religious experiences may still be of high importance for health for this numerically limited group in Sweden.

The significantly higher prevalence of religious service attendance among immigrants is notable. The age interval 18–34 years most likely contains many immigrants of Muslim, immigrant Christian, Catholic and other denominations. In contrast, the age interval 65–80 most likely contains a comparatively higher proportion of native Swedes who belong to the Lutheran Swedish church and the Swedish free churches (Puranen, 2020). This means that belonging to the Lutheran Swedish church and the Swedish free churches probably have the most important impact on the results of this study, because total mortality is highly concentrated in the age interval 65–80 (890 deaths in the age interval 65–80 and 10 deaths in the age interval 18–34 in 2008–2016).

The notion that organized religion and religious service attendance enhances health-promoting behaviors and practices (Nonnemaker et al., 2003) is supported by our results. The prevalence of low LTPA and daily smoking were significantly lower in the religious service attendance group compared to non-attenders. High-risk behaviors such as low LTPA and daily smoking may be one pathway behind the increased CVD mortality among non-attenders compared to religious service attenders.

SES is defined according to occupation, education and income. In this study, we defined SES by occupation and relation to the labor market because the 2008 survey contains no item concerning income and because the education item had a considerably higher number of missing. Still, it should be noted that recent studies have found significant associations particularly between educational level and social behaviors such as compliance with COVID-19 mandates as their main result (Maleki et al., 2022). In the present study, the proportion of high and medium non-manual employees was significantly higher among respondents who attended religious service during the past year than among respondents who did not. A reverse association was observed for skilled and unskilled manual employees who were significantly overrepresented among respondents who did not attend religious service during the past year. These findings imply somewhat higher educational level among those who attended religious service during the past year.

Social systems function in different social dimensions, not only in traditional SES and immigrant versus native dimensions. Individuals also associate in social networks, social groups, communities, organizations in civil society and across geographic space in ways not yet fully understood. This complexity, which has recently been discussed in terms of scale, context and heterogeneity, is sometimes referred to as the “complexity of the social space”. The issue of religion and religious service attendance in Sweden is thus complex. It probably warrants multiscale methods including multilevel analyses for full understanding (Barreiro-Balsa et al., 2022). Each country in western, southern and eastern Europe probably entails its own “complexity of the social space”, and the scope to generalize findings even to other European countries is probably limited. It should also be noted that it has never been possible to define geographic patterns of religion, religious service attendance and belonging to the Lutheran Swedish church as opposed to free churches in terms of simple urban versus rural dimensions. Throughout the 19th and 20th centuries, the tradition within the Lutheran Swedish church was much stronger in southern Sweden from northernmost Scania and in the rest of Götaland as well as in the northernmost part of northern Sweden (Norrland). In contrast, middle Sweden (Svealand including Stockholm and Uppsala) and the southern and middle parts of northern Sweden (Norrland) had weaker traditions within the Lutheran Swedish church and also generally weaker religious traditions regardless of the urban-rural geographic dimension (Lindström, 2001).

It should be noted that religion may also have adverse effects on health for both individuals and social contexts under certain circumstances (Krieger, 2017). The “dark side of social capital” has been thoroughly investigated (Villalonga-Olives & Kawachi, 2017). Community social capital may for instance have positive effects only for community members with high institutional trust and generalized trust in other people, while other people living in a community may experience exclusion (Subramanian et al., 2002). In the US, some studies have suggested that certain broad political party and ideological affiliations are associated with “fundamentalism” (Pabayo et al., 2015). In Sweden, which according to the World Values Survey (WVS) is one of the most individualized and secularized countries in the world (Inglehart, 2018), religious service attendance is significantly and strongly associated with high generalized trust in other people in addition to being significantly associated with lower all-cause mortality before the inclusion of social participation. In the Swedish individualized and secularized setting, individual religious service attendance seems to have mainly beneficial associations with both generalized trust and health (survival). Generalized trust and other key aspects of social capital have mostly not been included in studies of religion and health. In a secularized society religion may provide good preconditions for high generalized trust in others by religious/social participation and by providing a common faith, common norms and common cultural references which serve to enhance mutual understanding.

### 4.1 Strengths and limitations

This study is a large, population-based and prospective cohort study. The 54.1% response rate is acceptable given response rates in other surveys in approximately 2008. The participant population was acceptably representative of the register population concerning sex, age, country of birth and education. No serious selection bias has been observed in connection with the 2008 survey (Lindström & Rosvall, 2018).

Dichotomies of social participation items have long been judged to be valid in Sweden (Hanson et al., 1997). Occupation, education and income are different dimensions of SES. While being moderately to highly correlated, these SES dimensions are not identical. The 2008 survey contains no income item. The education item included had comparatively high**er** numbers of internally missing. The LTPA question is acceptably valid with regard to golden standards including heart rate (monitoring), four-day whole-day calorimetry, and double-labelled water (Wareham et al., 2003). The smoking item is valid (Wells et al., 1998). Swedish register data are valid by international comparison. The CVD, cancer and other causes mortality categories are also broad, which makes the risk of misclassification smaller.

Relevant covariates/confounders such as age, sex, SES, country of birth, chronic disease, BMI, LTPA, daily smoking, alcohol consumption, generalized trust in others and social participation items were included in the multiple analyses in Table 2.

## 5 Conclusion

Religious service attendance during the past year was significantly associated with lower all-cause mortality compared to non-attendance until various forms of social participation were introduced in the final model. In this highly secularized population, the social network pathway between religious service attendance and mortality may be important. Lower CVD mortality contributed to a limited extent to this consistent pattern, because CVD mortality was significantly lower for religious service attendance in the multiple models until BMI and health-related behaviors were introduced. There were no significant associations between religious attendance and cancer and other cause mortality.

## Ethical statement

The present study was approved by the Ethical Committee (*Etikprövningsnämnden*) in Lund (No. 2010/343).

## Author statement

ML conceived the idea of the manuscript, wrote the manuscript and revised the manuscript. ML also participated in the statistical analyses. MP conducted the statistical analyses, and read and commented the original and revised versions of the manuscript.

## Declaration of competing interest

There are no conflicts of interest.

## Data Availability

The authors do not have permission to share data.
